# Fatty Pancreas: Disease or Finding?

**DOI:** 10.6061/clinics/2021/e2439

**Published:** 2021-02-16

**Authors:** Lucas de Lucena Simões e Silva, Matheus Santos de Sousa Fernandes, Eline Autran de Lima, José Tadeu Stefano, Claudia P. Oliveira, José Jukemura

**Affiliations:** I Departamento de Gastroenterologia (LIM 07), Divisao de Gastroenterologia e Hepatologia, Faculdade de Medicina, Universidade de Sao Paulo, Sao Paulo, SP, BR; II Programa de Pos Graduacao em Neuropsiquiatria e Ciencias do Comportamento, Departamento Educacao Fisica, Universidade Federal de Pernambuco, PE, BR; III Faculdade de Educacao Fisica, Universidade de Pernambuco, Recife, PE, BR; IV Departamento de Gastroenterologia Divisao de Cirurgia Digestiva, Instituto do Cancer (ICESP), Hospital das Clinicas HCFMUSP, Faculdade de Medicina, Universidade de Sao Paulo, Faculdade de Medicina, Sao Paulo, SP, BR

**Keywords:** Fatty Pancreas, Obesity, Physical Activity, Steatosis, Metabolic Syndrome

## Abstract

Despite a growing number of investigative studies on pancreatic fat deposition, there remains no clear indication regarding the clinical relevance of fat infiltration in the pancreas, also called fatty pancreas (FP). An individual’s body weight is correlated with their pancreatic weight. Moreover, lipid infiltration causes disorders that compromise not only morphology but also metabolic functions. Fat infiltration leads to insulin resistance, type II diabetes mellitus, and pancreatic cancer; however, knowledge about pancreatic fat content and aspects related to the clinical profile remains unclear in the literature. The present review describes the current knowledge of FP, including its pathophysiology and clinical implications, as well as lifestyle changes in FP.

## INTRODUCTION

Schaefer (1) first reported the excessive accumulation of fat and a correlation between the body and pancreatic weight. A decade later, Ogilvie (2) reported a 17% higher pancreas weight in obese cadavers compared to that in lean cadavers, corroborating Schaefer’s results. Olsen (3) observed a significant increase in pancreatic fat with advancing age in both sexes, and Stamm (4) reported that a 25% increase in pancreatic fat was responsible for a significant increase in the frequency of type II diabetes mellitus (DM2) and atherosclerosis.

Since the first report, many terms have been used to describe intrapancreatic fat (IPF) (5,6). There is no standardized nomenclature for this phenomenon; however, the term non-alcoholic fatty pancreas disease (NAFPD) has gained acceptance.

NAFPD is described as a pancreatic steatosis phenotype in the absence of alcohol consumption, viral infections, toxins, or congenital metabolic syndromes and is associated with insulin resistance, malnutrition, obesity, metabolic syndrome, increasing age, pancreatic fibrosis (6,7), and pancreatic cancer (8). Although NAFPD is often compared to non-alcoholic fatty liver disease (NAFLD), which has been widely evaluated, current knowledge on NAFPD is still under development, despite the recent increased interest (9-12). Aspects related to its clinical significance and their relationship with chronic pancreatitis have not been fully elucidated. The purpose of this review is to describe the current knowledge of fatty pancreas (FP), including its pathophysiology and clinical implications, as well as the possible beneficial effects of lifestyle changes in FP.

### Prevalence

Since its discovery in 1933 (1), NAFPD has not been widely evaluated. Its epidemiology is not well defined because of the lack of parameters for clear diagnosis and differences in terminology. FP is generally considered a random finding during abdominal imaging tests performed for other reasons.

Due to the lack of standardized screening tests, epidemiological data on NAFPD are limited. In the Asian population, the prevalence of FP may be between 16% and 35%. However, data are lacking in Western populations. An epidemiological study by Pham et al. (13) investigating FP in a pediatric population reported a prevalence of 8% and 19% in non-obese and obese children, respectively. In addition, pancreatic steatosis was significantly associated with NAFLD. However, the study evaluated only hospitalized children and not the general population, which is a limiting factor in assessing the real prevalence of NAFPD in this population. Another study in a Western population by Sepe et al. (14) evaluated 230 adults in different age groups. Approximately 28% of the evaluated population had FP and 23% had fatty liver. Patients aged 41-70 years were more susceptible to FP and the presence of any component of metabolic syndrome (MS) was responsible for the 37% increase in the prevalence of FP.

Central obesity is becoming a serious public health problem among all age groups; therefore, an increased incidence of NAFLD and NAFPD is expected. Besides obesity, some studies have reported an association between NAFPD and NAFLD (15-17). Approximately 50-80% of patients with NASH have FP, as confirmed through abdominal imaging examinations (18,19). These results showed that the presence of NAFLD may be a risk factor for NAFPD. Therefore, patients affected by MS must be evaluated for both NAFLD and NAFPD; however, more epidemiological studies are needed to better understand NAFPD.

### Physiopathology

Two main mechanisms may explain the accumulation of fat in pancreatic tissue. Fatty replacement is characterized by the death of pancreatic acinar cells and their replacement by adipocytes, and fatty infiltration, which is characterized by the accumulation of fat in the pancreas and association with MS and/or obesity, which defines NAFPD.

“Fatty replacement” is characterized by the death of pancreatic acinar cells and their respective replacement by adipocytes, which can be caused by several conditions. The main factors for pancreatic cell death include genetic factors such as fibrosis or clinical features such as excessive alcohol consumption, viral infections, iron overload (mainly represented by hemochromatosis), use of medication (corticosteroids), or obstruction of the pancreatic duct (chronic obstructive pancreatitis).

“Fatty infiltration” occurs when there is an infiltration of adipocytes in the pancreatic tissue. Obesity is the main contributing factor for this condition, leading to NAFPD (14,20). Adipose tissue is considered an endocrine organ because it sends signals to other organs. During weight gain, the storage of adipose tissue is exceeded, causing a redistribution of fat in non-adipose tissues such as the liver, skeletal muscle, and pancreas (21). The infiltration of pancreatic fat initially causes hypertrophy and hyperplasia of the pancreatic cells (22), resulting in clinical conditions such as insulin resistance, β-cell dysfunction, and DM2 (23).

The pancreatic weight of obese mice with leptin deficiency was elevated compared to that in lean mice of the same lineage; moreover, obese mice also showed high amounts of intralobular and fat content and high levels of pro-inflammatory cytokines (tumor necrosis factor [TNF]-α and interleukin [IL]-1b). In addition, greater development of FP, pancreatic inflammation, pancreatic fibrosis, and insulin resistance was observed in mice exposed to high-fat diets (24). Indeed, the pancreas seems to be more susceptible to fat deposition than the liver, with 15 weeks of high-fat diet in mice resulting in a 5x greater fat accumulation in the pancreas compared to that in the liver (25).

Human pancreatic fat content has been associated with increased insulin resistance, MS, and hepatic fat content (16). Moreover, increasing age has been associated with pancreatic steatosis. Saisho et al. (26) reported that pancreatic fat content increased with age throughout childhood and reached a plateau until 50 years. Multiple regression analysis showed a pancreatic lipid content of 59.2% by magnetic resonance imaging (MRI). This finding may vary according to sex and visceral adipose tissue (27). Men have a higher pancreatic fat content than women and the highest prevalence of NAFPD at 40-49 years of age. Moreover, the prevalence of NAFLD in women is very low until menopause (27,28). Taken together, these results suggest that aging and hormonal changes are related to the emergence and development of NAFPD, although further confirmatory studies are needed.

### Fatty Pancreas and Associated Diseases

Studies have suggested that fatty infiltration of the pancreas leads to loss of β cells and their function, which has been identified as the main factor in the development of DM (20). The most frequent explanation for NAFPD and pancreatic β cell dysfunction is glycolipotoxicity. Hyperglycemia causes the inhibition of the mitochondrial β-oxidative process in β cells, through a cascade of enzymatic processes, ultimately leading to the intracellular accumulation of triglycerides. Insulin resistance simultaneously reduces insulin inhibitory activity in lipolytic processes and leads to increased levels of free circulating fatty acids (29).

Wu and Wang (30) observed an association between the incidence of FP and metabolic features (Table 1). Their results suggested that older age, high body mass index (BMI), increased waist circumference, and metabolic variables (glycated hemoglobin, biochemical profile, and systolic blood pressure) were associated with FP, that is, factors that are related to the onset of MS (31). Tushuizen et al. (32) evaluated pancreatic fat content and β-cell function in men with and without diabetes mellitus II (Table 1), showing a mean pancreatic fat content in diabetics compared to that of controls of 20.4% *versus* 9.7%, respectively. In addition, pancreatic fat was negatively correlated with β-cell function parameters. Patients affected by MS are recommended to undergo a more specific investigation to prevent FP. In addition to MS, it is also important to understand the impact of DM on the pancreas because it is the most common manifestation of diabetes and has a high incidence in this population (23,26).

In humans, there is a divergence in findings. Saisho et al., (26), (Table 1) did not observe an association between pancreatic steatosis and β-cell dysfunction in patients with established DM, however, Chai et al., (33), reported an association between pancreatic steatosis and DM2. Van Der Zijl et al. (34) showed higher levels of pancreatic fat in patients with pre-diabetes but observed no relationship between FP and insulin secretion. Table 1 presents a summary of studies on non-alcoholic fatty pancreatic disease and its associations with different comorbidities.

### Fatty Pancreas and Pancreatic Cancer

Central obesity has been associated with different types of cancers, including pancreatic cancer. As pancreatic steatosis is related to obesity, FP may be related to the development of pancreatic cancer. Therefore, it is important to understand the role of obesity in the development of pancreatic cancer because of its complications. Stolzenberg-Solomon et al. (8) reported a 45% increased risk of pancreatic cancer in patients with a BMI ≥35 kg/m^2^. In addition, a high waist circumference measurement in women was associated with an increased risk of pancreatic cancer. An epidemiological study by Arslam et al. (35) showed similar results in an evaluation of approximately 4400 adult patients, showing a positive association between high BMI and the risk of pancreatic cancer in both sexes. Moreover, women with the highest BMI values were more susceptible to cancer than women with age-appropriate BMI values. A clinical trial showed that an excess of total energy consumption increased the incidence of pancreatic cancer (36). In addition, there is evidence that the risk of pancreatic cancer is higher with increasing BMI, with obese individuals having a 19% higher risk compared to individuals with normal BMI (37,38).

### Fatty Pancreas, Physical Activity, and Exercise

Assuming that FP develops through fat accumulation and infiltration in the pancreas (as in other organs), lifestyle may be one of the determining factors for the development or regression of this disease.

Stolzenberg-Solomon et al. (8) evaluated the associations between related pancreatic cancer risk factors and regular physical activity (Table 2), showing that higher BMI values were directly related to pancreatic cancer and its risk factors in both men and women. While no significant differences were observed for physical activity and decreased risk of pancreatic cancer, this result is questionable. During the analyses of physical activity level the patients were not separated according to their respective BMI; rather, they were only categorized according to the amount of physical activity, related intensity, and the metabolic equivalents (METs) (8). Indeed, individuals with a regular BMI may interfere in the analysis and results of FP and pancreatic cancer risk factors. Michaud et al. (38) reported no association between pancreatic fat content and risk factors associated with pancreatic cancer in this population; however, this association was observed in overweight individuals.

Hannukainen et al. (39) showed that physical fitness had no significant effect on pancreatic fat content, however, they reported lower values of pancreatic fat content in physically active individuals compared to their monozygotic twins (Table 2). Although a difference was observed in the levels of physical fitness between the twins, based on their BMI they were considered healthy or slightly overweight. These results corroborate those reported by Michaud et al. (38), in which no associations between pancreatic fat content and regular physical activity were found among individuals with regular BMI.

e Silva et al. (40) evaluated the effects of an 8-week aerobic exercise protocol on genes related to insulin resistance and inflammation in the pancreas of ob/ob mice with NAFLD (Table 2). Their findings showed that aerobic exercise attenuated body weight gain and food intake and generated positive effects on the expression of genes related to insulin resistance and inflammation in both the liver and pancreas. In addition, histopathological analysis, showed a slight decrease in pancreatic inflammation in the trained animals, although the difference was not statistically significant.

Although some studies have presented relevant data on lifestyle and factors related to FP, there remains no consensus in the literature about specific physical training for this disease. However, it is recommended that patients have healthy eating habits and increase regular physical activity. Thus, reducing pancreatic fat content through lifestyle changes may benefit the health status and clinical profile of patients with FP. Table 2 presents a summary of studies that evaluated the possible effects of regular physical activity and exercise in NAFPD.

## CONCLUSION

FP is an increasingly recognized disease entity and is strongly associated with comorbidities including DM, obesity, dyslipidemia, MS, liver fat content, acute pancreatitis, and, in some cases, pancreatic cancer.

Although few epidemiological studies have reported on the prevalence and natural history of this condition, further studies on FP are expected due to its strong association with central obesity and MS.

Studies evaluating physical exercise effects and the associated features of FP and NAFPD are still under development and require further investigation to improve understanding. However, regular physical activity should be encouraged in patients with this condition as exercise increases caloric expenditure and basal metabolic rates which positively influences clinical and biochemical factors.

## AUTHOR CONTRIBUTIONS

e Silva LLS and Fernandes MSS were responsible for the study conception and design, data search, and manuscript writing. Lima EA was responsible for data search, organization, and manuscript writing. Stefano JT, Oliveira CP and Jukemura J were responsible for data analysis and interpretation, content review, and final approval of the manuscript to be published.

## Figures and Tables

**Table 1:** Characteristics of studies evaluating pancreatic-associated diseases included in the present review.

Author	Pancreatic evaluation method	Sample size (n)	Age (years)	Main findings
Chai et al. (33)	MRI	70	16-72	Higher average pancreatic fat content in DM2; Uniform distribution of fat content was uniform in lean and DM2 groups
Saisho et al. (26)	CT-scan	2021	0-100	Overweight and obese individuals have 16% and 32% more pancreatic fat content respectively; Correlation between pancreatic volume and BMI in patients aged 20-60 years
Tushuizen et al. (32)	MRI	36	35-65	Impaired β-cell function and increased pancreatic fat content in diabetic patients; Elevated hepatic fat content and association between fasting plasma glucose and HOMA-IR
Van Der Zijl et al. (34)	MRI	64	47-63	Inverse association between pancreatic fat content and insulin sensitivity; Pancreatic fat content increases gradually with glucose metabolism impairment
Wu and Wang (30)	AUS	557	38-63	Fatty pancreas associated with age, hyperglycemia, hyperlipidemia, and fatty liver; Association between fatty pancreas and metabolic syndrome components

MRI: magnetic resonance imaging; CT: computed tomography; AUS: abdominal ultrasonography; HOMA-IR: homeostatic model assessment of insulin resistance; DM2: type ll diabetes mellitus.

**Table 2:** Characteristics of physical activity/exercise related to the features of fatty pancreas in the present review.

Author	Sample Size (n)	Physical activity/Exercise modality	Physical activity evaluation method	Main findings
e Silva et al. (40)	14	Aerobic	Running capacity treadmill test	Improvements in IRS-2, GLUT-2, IL-6, and IL-10 in both the liver and pancreas. Lower inflammatory infiltrate values on histopathological analysis in the training group.
Hannukainen et al. (39)	16	Aerobic	Questionnaire	Less visceral and subcutaneous fat. Lower values of HOMA indexes, apolipoprotein A1, and hepatic fat content and discrete decrease in pancreatic fat in more active individuals.
Michaud et al. (38)	163,689	Aerobic/resistance	Questionnaire	Lower physical activity levels were associated with higher BMI values; Individuals with BMI ≥30 kg/m^2^ had a 72% higher risk of pancreatic cancer; Moderate physical activities such as walking, running, and/or hiking were associated with a 54% reduced risk of pancreatic cancer when practiced at least 4 h/week compared to 20 min/week.
Stolzenberg-Solomon et al. (8)	495,035	Aerobic/resistance	Questionnaire	Physical activities spent as leisure and/or work, at least 5X/week were inversely associated with lower BMI; Severe obesity was associated with a higher risk of pancreatic cancer in both men and women; waist circumference was associated with risk of pancreatic cancer only in women

BMI: body mass index; GLUT-2: glucose transporter; HOMA: homeostatic model assessment; IL-6: interleukin 6; IL-10: interleukin 10; IRS-2: insulin receptor substrate 2.
